# Workplace Violence Experiences Among Nurse Educators in Saudi Arabia: A Qualitative Descriptive Study

**DOI:** 10.1002/nop2.70769

**Published:** 2026-08-21

**Authors:** Jordan Tovera Salvador, Sana Abdulkareem Almahmoud, Reem Nasser Mohammed Al‐Dossary, Basim Muzil Mohammad Al‐Anazi, Friyal Mubarak Alqahtani, Areej Al‐Otaibi, Ola Ibrahim Ramzi, Neama Mohamed Kamel, Faisal Lafi Aldhafeeri, Maria Abigail Trinidad, Ahrjaynes Balanag Rosario, Abdulla Mohammed Ibrahim, Suzette Golez Cabonce, Darwin Damsani Agman, Eshtiaq Abdulaziz Alfaraj

**Affiliations:** ^1^ College of Nursing Imam Abdulrahman Bin Faisal University Dammam Eastern Province Saudi Arabia; ^2^ Deanship of Quality and Academic Accreditation Imam Abdulrahman Bin Faisal University Dammam Eastern Province Saudi Arabia; ^3^ College of Applied Medical Science Imam Abdulrahman Bin Faisal University Dammam Eastern Province Saudi Arabia

**Keywords:** aggression, bullying, incivility, nursing education, Saudi Arabia, teaching, universities, violence, workplace violence

## Abstract

**Background:**

Over the past decade, few studies have explored workplace violence (WPV) in nursing education in the Kingdom of Saudi Arabia. Understanding nurse educators' experiences of WPV is critical to craft effective and efficient interventions. The study explores nurse educators' experiences with WPV across multiple nursing educational settings in Saudi Arabia.

**Methods:**

Twenty‐five nurse educators were interviewed using a semi‐structured interview guide at various nursing schools (colleges and universities) in the Eastern Province of Saudi Arabia. The COREQ criteria were followed to report the qualitative findings of participants' narratives.

**Result:**

Three themes were generated based on educators' experiences of WPV: (1) the workplace violence aggression; (2) the keys to successful adaptation; and (3) the recommendations for the future. A lack of awareness about the nature of WPV is the primary reason for its presence in academic settings. Awareness campaigns are needed to promote active engagement and sustainable actions to combat and prevent it in nursing education facilities in Saudi Arabia.

**Implications for the Profession and/or Patient Care:**

The findings of this research would serve as a catalyst for nursing advocates, experts, and academic leaders to develop innovative approaches to eliminating or reducing WPV recurrence, such as strong preventive policies and responsive actions, confidential reporting, clear anti‐WPV protocols, simulation‐based training, leadership accountability, improved peer and mental health support, and ongoing monitoring systems.

**Patient or Public Contribution:**

No patient or public contribution. Nurse educators participated as interviewees.

## 1 Introduction

Workplace violence (WPV) is a common phenomenon worldwide, not only experienced by healthcare professionals (e.g., nurses, doctors) but also in various professions that require interpersonal communication, such as hospitality management and customer service (Abdelmalik et al. 2025). Various studies identified that nursing is one of the occupations with the highest violence incidence, compromising nurses' well‐being and work stability. Similarly, other studies often link WPV to burnout syndrome and attrition (Yang et al. 2025; Liu et al. 2019; Han et al. 2022; Tsukamoto et al. 2022; Rayan et al. 2019). However, limited studies have shown WPV in nursing education. In fact, in a qualitative systematic review conducted by Park and Kang (2022) focusing on faculty‐to‐faculty incivilities, notably, all studies included originated in Western contexts, and authors emphasized the need for Eastern‐focused research. Another systematic review on organizational bullying among nursing faculty's consequences, contributing factors, and interventions revealed serious challenges, harming both individuals and institutions. Thus, effectively tackling this issue demands comprehensive, evidence‐based strategies—such as structural reforms, leadership development, and psychosocial support. Therefore, academic institutions must foster safe, inclusive, and respectful environments to retain faculty and uphold nursing education quality (Zeydani and Atashzadeh‐Shoorideh 2026).

Goldberg et al. (2013) and Fuller (2019) also mentioned that social bullying in nursing academia had become too prevalent, which led faculty members to lose their happiness, passion, and teaching satisfaction. These had resulted in them leaving the profession feeling disrespected and marginalized. Yosep et al. (2024) and Bambi et al. (2018) discussed the relationship between WPV and social bullying. The latter serves as a foundational psychological factor and an early indicator of broader workplace violence within nursing education. It operates as a sustained pattern of horizontal or vertical aggression, gradually normalizing toxic behaviours in any academic settings. As a matter of fact, a psychometric development of an instrument measuring social bullying in nursing education was created to possibly obtain the differences between bullying and incivility that later can turn out to be WPV. This process not only fosters a culture of incivility but also escalates towards more severe psychological and professional harm. Ultimately, social bullying bridges the continuum from minor incivility to significant WPV, impacting both faculty and students as they move into clinical practice (Beitz and Beckmann 2021). Underreporting and a lack of serious consideration for reporting mechanisms have perpetuated its acceptance as a cultural norm within organizations; thus, higher rates of reported bullying behaviours, particularly horizontal violence and unsupportive leadership, are strongly correlated with nurse educators' increased intent to leave the academic field. This highlights how disruptive work environments are a significant driver of faculty attrition and pose critical risks to retention in nursing education (Tyler et al. 2022).

In Saudi Arabia, WPV has been reported as a major issue concerning the healthcare organizations especially in various fields of the nursing profession, which necessitates open discussion to devise preventive measures to counter its prevalence (Aljohani 2021). Abdelmalik et al. (2025) reported a recent study of a systematic review and meta‐analysis, emphasizing the high prevalence of WPV in all nursing settings (nearly 62%) in the kingdom. As a matter of fact, there is limited research on WPV in nursing education in Gulf Cooperation Council (GCC) countries. The existing literature highlights a significant gap, with few empirical studies conducted within the region's academic nursing institutions. More focused research is needed to thoroughly understand and effectively address WPV among nursing educators in GCC countries. There are few literatures and studies that were found focusing on the following: (1) nursing students from the perspectives of clinical facilitators or preceptors (Dafny et al. 2025), but not from the perspectives of the nurse educators in the educational facilities; (2) faculty incivilities in nursing schools (multi‐country study including Saudi Arabia; Park and Kang 2022); (3) a descriptive cross‐sectional study on incivility, job satisfaction, and intent to leave among Saudi nursing faculty (Alsallum 2025); (4) a systematic review on nursing workforce challenges in GCC countries (Ansari et al. 2026); and (5) a systematic review and meta‐analysis of the prevalence of WPV among nurses in Saudi Arabia, suggesting that such behaviours are significant within clinical and potentially academic nursing contexts. Likewise, the majority of WPV published works bounded to insights and strategies as a method minimize its occurrence by introducing WPV as a topic within curriculum delivered in the form of simulated experiences; projected to faculty and students within the code of conduct to guide their behaviours where it exhibited via role modelling (Sanner‐Stiehr and Ward‐Smith 2017); and utilizing prevention management program from nursing organizations (International Council of Nurses 2009).

Consequently, a previous qualitative study was conducted by the primary author exploring the experiences and insights of nurse educators in one government university in the Eastern Region of Saudi Arabia resulting in a WPV model called “Triple‐A: Acquaint, analyse, and act”, which provides a call‐to‐action for nurse managers and leaders to create sustainable creative and innovative strategies to educate and combat any form of violence in the nursing profession at‐large (Salvador 2022). However, the current study assessed other nursing schools across the region with a total of eight colleges and universities, excluding the previous research locale. This large‐scale qualitative study, initiated by a single university, enhanced generalizability by reducing institutional bias, increased the relevancy of findings, and enabled identification of more comparative insights, differences, and patterns of WPV, and unique challenges or strengths across institutions. Nevertheless, there was no overlap with previously published qualitative research. Moreover, the interview guidelines, datasets, analytic methodologies, and research dissemination are entirely independent. Although both studies examined nurse educators' experiences of WPV in Saudi Arabia, this study employed different methodological frameworks. It examined additional characteristics that were not investigated in the previous primary research. Therefore, region‐wide data and results are more significant for policymakers and stakeholders, supporting changes that benefit nursing education institutions across the region.

Furthermore, most studies published in the kingdom have focused primarily on healthcare settings, specifically clinical units within hospitals, primary care centers, and nursing home care (Alyaemni and Alhudaithi 2016; Alsmael et al. 2020). Moreover, in a qualitative study conducted by Dillon (2012) at a government hospital, registered nurses reported encountering various forms of WPV, which were categorized into three themes and were presumably factors of WPV: (1) coworkers exhibiting unjust and violent behaviour; (2) socio‐cultural differences towards healthcare workers; and (3) external influences causing WPV. In a study conducted in the kingdom, the awareness of 210 healthcare providers about training in using reporting system revealed 31.4% lacked knowledge on how to report violent cases, while 68.6% lacked training (Towhari and Bugi 2020). Simultaneously, it investigated the influence of the latest health liabilities and punishments for WPV, legislated by the Ministry of Health (MOH) of Saudi Arabia, and reported a nearly 20% reduction in cases since their implementation (Towhari and Bugi 2020). Despite numerous attempts to eliminate this increasingly social phenomenon, the search for the most comprehensive, cost‐effective, robust, and systematic anti‐WPV program continues, which is open to collaborative efforts and initiatives.

Therefore, this qualitative study aims to contribute to the literature on WPV issues in nursing education to provide a deeper understanding of the various forms of violence that nurse educators may experience in the present and in the future. Therefore, this investigation analysed the narratives of the selected participants to answer the grand tour question, “What are the experiences of nurse educators towards WPV in Saudi Arabia?”

## 2 Methods

### 2.1 Design

A qualitative descriptive design was pursued as the most appropriate approach to unfold the phenomenon under investigation, which may affect nurse educators' experiences within academic settings, effectively revealing nursing education issues that require attention for prevention and proposing concrete recommendations for improvement and eradication (Creswell and Creswell 2023; Polit and Beck 2014). Likewise, the qualitative approach helped answer questions about what, who, when, and where the phenomenon is experienced or occurs (Lim 2025).

### 2.2 Participants and Setting

Using a purposive sampling method, 25 participants (ten men and 15 women) were recruited based on specific inclusion criteria. The participants were volunteer nurse educators and/or faculty members and teaching staff of reputable colleges and universities offering nursing programs in the Eastern Region of Saudi Arabia, ranked as demonstrators, lecturers, or professors; they hold a PhD, master's degrees, and bachelor's degrees, and with at least 5 years of working experience in the academic settings to ensure familiarity with the organizational climate and culture, willing to share their personal experiences of WPV and able to converse in English. The participants had a minimum of 10 to a maximum of 22 years of experience in nursing education, particularly in the colleges and universities. The participants' age range was 35 to 60 years old. Table 1 displays the participants' socio‐demographic data.

**TABLE 1:** Sociodemographic data of participants.

No.	Code	Gender	Age	Nationality	Civil status	Education	City in the region	Academic rank	Experience
1	Australia	F	37	Saudi Arabia	Married	Master	Hafar Al‐Batin	Lecturer	11
2	Brazil	M	56	Jordan	Married	Doctoral	Al Khobar	Professor	16
3	China	F	38	India	Married	Master	Al‐Ahsa	Lecturer	13
4	Denmark	F	57	Sudan	Married	Master	Al‐Ahsa	Professor	19
5	Egypt	M	44	Philippines	Married	Master	Dammam	Lecturer	13
6	Finland	F	56	Egypt	Married	Doctoral	Al Khobar	Professor	18
7	Gambia	M	36	Philippines	Single	Doctoral	Dhahran	Professor	10
8	Honduras	F	37	Saudi Arabia	Married	Master	Hafar Al‐Batin	Lecturer	17
9	India	F	58	Egypt	Married	Doctoral	Al‐Ahsa	Professor	21
10	Jordan	F	45	India	Married	Master	Dhahran	Lecturer	12
11	Kuwait	F	43	India	Married	Master	Dammam	Lecturer	13
12	Lebanon	F	35	Saudi Arabia	Married	Bachelor	Dhahran	Demonstrator	15
13	Maldives	M	46	Jordan	Married	Master	Dhahran	Lecturer	16
14	Netherlands	F	57	Egypt	Married	Doctoral	Dammam	Professor	21
15	Oman	M	52	Philippines	Married	Bachelor	Al‐Ahsa	Demonstrator	18
16	Philippines	M	41	Jordan	Married	Master	Dammam	Lecturer	16
17	Qatar	F	40	Saudi Arabia	Married	Doctoral	Hafar Al‐Batin	Professor	17
18	Russia	M	41	Jordan	Married	Master	Al Khobar	Lecturer	15
19	Singapore	F	53	Philippines	Married	Master	Dhahran	Lecturer	13
20	Thailand	M	38	Philippines	Single	Master	Al Khobar	Lecturer	14
21	Uruguay	F	42	Saudi Arabia	Married	Doctoral	Hafar Al‐Batin	Professor	15
22	Vietnam	M	39	Saudi Arabia	Married	Master	Al Khobar	Lecturer	10
23	Wales	F	60	Egypt	Married	Doctoral	Dammam	Professor	22
24	Yemen	M	37	Jordan	Single	Master	Al‐Ahsa	Lecturer	11
25	Zimbabwe	F	59	Saudi Arabia	Married	Doctoral	Hafar Al‐Batin	Professor	21

Furthermore, participants employed in universities and colleges in the Eastern Province of the Kingdom of Saudi Arabia that offer nursing education programs, particularly those with national and international certifications, are considered centers of excellence in nursing education. In contrast, most settings feature a diverse group of multicultural nurse educators from around the world, including Saudi Arabia, Jordan, India, Sudan, the Philippines, and Egypt. However, 17 participants speak native Arabic and graduated from Australia, Canada, the United Kingdom, and the United States of America, providing exposure to diverse cultures and academic backgrounds.

### 2.3 Data Collection

Individual semi‐structured interviews were conducted from September 2023 to February 2024 with prospective participants who satisfied the inclusion criteria. However, prior to the interviews, individual briefing sessions were held to explain the aim, objectives, data collection, and essential protocols for the study. To meet the COREQ reporting standards and further enhance methodological transparency and qualitative rigour, the researchers considered the following essential elements of the semi‐structured interview guide as per Dikko (2016): (1) three participants were invited for preliminary interviews lasting from 20 to 40 min to ensure clarity and content validity, demonstrating that necessary conceptual questions were focused on answering the core research accurately and ambiguous questions were reworded to make them easier for participants to understand; (2) debriefing sessions were conducted with the participants, written comments initiated and audio recordings evaluated; (3) detail revisions: the researchers clearly listed changes made to the interview guide based, including rephrasing questions for clarity, adding new prompts, and removing ambiguous and redundant items for logical sequence and smoother transitions; (4) version control: the primary researcher maintained several edited versions of the interview guides and used the final approved guide in the main study in which probing prompts were added; and (5) all researchers affirmed that these steps were taken to bolster the audit trail and support the qualitative rigour of the research in which the language was unified to be more neutral and understood by participants from diverse backgrounds. These adjustments have enhanced the interview guide used and made it ready for data collection. Table 2 shows the interview guide used in this qualitative investigation.

**TABLE 2:** Interview guide.

Can you introduce yourself? Can you elaborate on your role as a nurse educator or teaching faculty in the college? Can you describe your workplace environment? Can you expound on what you know about WPV? Can you share your experiences and perspectives on WPV? How does WPV affect you personally and professionally? How does your organization handle WPV? What do you think can be the most effective and efficient way to eradicate WPV? How do you perceive WPV in the nursing education sector 10 years from now? What are your final thoughts about WPV in nursingeducation?

Furthermore, to ensure researchers' adherence to the qualitative research approach, several sessions focused on qualitative research methods, interviewing skills, transcriptions, coding, content analysis, and manuscript writing were held. The primary researcher assigned five researchers to conduct the individual interviews. The sessions were scheduled based on participants' availability, preferred timing, and designated locations where they feel comfortable sharing their experiences (e.g., offices, faculty lounges, or cafes). The interview sessions lasted 30–50 min, and the number of sessions depended on the quality of the interview data, with no interview exceeding to four sessions. Probing and follow‐up questions were used to ensure understanding and avoid any ambiguities. In addition, the participants were debriefed on the meaning and insights gained before the interview ended. All interview sessions were audio‐recorded. Lastly, field notes and a personal reflective journal were written after each interview.

### 2.4 Data Analysis

During data gathering, the audio recordings were listened to and later transcribed to determine data sufficiency. New information and insights were assessed iteratively through multiple successive interviews to help researchers determine data saturation. Therefore, the dataset on the phenomenon under study was considered sufficiently rigorous to proceed with qualitative content analysis (QCA) when no relevant and significant statements were identified in the nurse educators' narratives. This step added legitimacy and reliability to the research findings, ensuring that data were obtained based on evidence‐based judgement (Williamson and Johanson 2018; Erlingsson and Brysiewicz 2017). Four members of the research team evaluated the transcripts after each round of individual interviews to unveil emergent themes, patterns, and categories from the smallest segment of content to be analysed (e.g., word, phrase, sentence, paragraph, idea). These semantics were meticulously identified using MS Excel spreadsheets based on the developed coding scheme (coding frame), in which clearly defined categories and code definitions were established (e.g., Workplace Aggression: Any behaviour designed to cause psychological or physical harm to a colleague or organization).

Additionally, to ensure comprehensiveness and clarity, the team used the preliminary coding scheme on a sample of the data and revised it based on ambiguities, overlaps, or missing categories. Later, the team systematically finalized the coding scheme for the entire dataset, assigned codes to text segments that match the definitions in the coding frame, and utilized qualitative data analysis through a manual method, which offered deep immersion with data and significant cognitive connection, fewer distractions, flexibility, and simplicity (fluid boundaries). The primary researcher led the team in checking coding consistency and reliability by assigning the team to work in groups to assess inter‐coder reliability, discuss any ambiguities and discrepancies, revisit the coding scheme, recode if necessary, and collectively refine the finalized codes for accuracy and consistency. To resolve discrepancies, the team held a series of roundtable discussions to build consensus and consulted a senior expert within the team to resolve disputed segments, break any ties, and reach a consensus. Analytic notes and codebooks detailed this approach. Throughout the coding process, four criteria were observed based on Tracy (2020) to develop meaningful and conceptual categories: (1) direction (information), (2) intensity (strength), (3) space (message size), and (4) frequency (occurrence). After finalizing the results, the researchers examined the coded data for patterns, themes, and their relationships. Furthermore, the final categories and themes underwent an extensive literature review to stimulate discussion. It helped unveil participants' experiences, offering valuable insights into WPV in the academic sector and providing meaningful recommendations to mitigate this growing issue.

### 2.5 Researcher Positionality

The research team consisted of 11 assistant professors (three male and eight female), three lecturers (two male and one female), and one male demonstrator. The researchers had different nationalities: the Philippines (five), Saudi Arabia (seven), Egypt (two), and Jordan (one), with extensive experience in education, quality management, and research. Likewise, all researchers work at one university but in three different colleges and deanships: Nursing (thirteen), Quality and Academic Accreditation (one), and College of Applied Medical Science (one). These details were transparently disclosed to the participants during the first interview session to build rapport and balance the power dynamics. The topic is fitting to the present state of the research team, as they are all expert educators in their field. This positionality element may introduce a potential researcher's bias, as their status may influence their observations, credibility, and reflexivity in qualitative research. Accordingly, it may tarnish the trustworthiness of the findings since it affects how the data were evaluated and presented. Researchers address this major challenge through reflexivity, a practice in which researchers note their daily personal feelings and views to track changes in choices during the study and continually reflect on their views and biases with each other that may influence interactions with participants and the findings derived from those interactions. Moreover, to ensure that the researchers' assumptions did not impact the data analysis and interpretation of the study findings, participants' validation at the end of each interview, keeping a reflective journal, and employing peer debriefing to evaluate interpretations were utilized.

### 2.6 Scientific Rigour

The scientific rigour of the dataset was ensured by thoroughly examining the qualitative procedure (Johnson et al. 2020). The research team upheld the fundamental principles of trustworthiness throughout the entire process: The following measures were taken for reliability purposes: (1) *Credibility*—audio recordings were transcribed some hours later following each interview, and the transcriptions were compared with interviews and journal notes of the interviewer; (2) *Confirmability*—the researchers invited four professors to confirm and validate the results obtained after analysing the data in many rounds of discussion; (3) *Dependability*—full documentation was kept as to be able to audit the procedures used, and the conclusion drawn was based on the stories told by participants in transcripts; and (4) *Transferability*—three nurse educators in GCC countries were invited to validate results. Additionally, participants also contributed to validating the results by reviewing the transcribed narrative accounts before the dissemination of the findings. The COREQ guidelines were used to report qualitative results (Tong et al. 2007).

### 2.7 Ethical Approval

The study's ethical approval was sought from the Imam Abdulrahman Bin Faisal University Institutional Review Board (IRB‐2023‐04‐136) on 22 March 2023. The researchers observed bioethical principles throughout the study procedures to safeguard participants' rights, reduce and mitigate the risk of harm, elevate scientific validity, and maintain global ethical standards. The study adhered to the Declaration of Helsinki 2013 (World Medical Association 2013). Hence, an introductory debriefing session for participants was conducted, covering the study's aim, data collection methods, and participation, benefits, and risks prior to the data collection stage. To maintain participant de‐identification, each participant was assigned an alphabetical country code (Participant 1 = Australia). The researchers observed the bioethical principles throughout the study procedures. After each scheduled interview, a debriefing session was conducted to affirm understanding of the meaning gained; it also provided an opportunity to ensure participants' well‐being after discussing such a topic‐sensitive topic. The university's resident psychologist received referrals from participants experiencing emotional turmoil and upheaval for appropriate counselling and interventions. Furthermore, participants were reassured that they could withdraw from the interview at any time without explanation if they felt uncomfortable. Finally, the transcribed verbatim and audio recordings were kept in a password‐secured cabinet and will be destroyed 5 years after the date of publication.

## 3 Results

The nurse educators' narratives about WPV yielded nine categories, leading to the three emergent themes: (1) “the workplace aggression experiences”; (2) “the keys to successful adaptation”; and (3) “the recommendations for the future.” Table 3 summarizes each emerging theme, including its descriptions, categories, and keywords/phrases.

**TABLE 3:** Emergent themes, meaning, categories, and keywords/phrases.

Keywords/Phrases			Categories	Emergent theme	Meaning
Bullying Assault Poor image Ageism Socio‐cultural diversity Lack of professional experience Physical Abuse Mobbing Sexism in the nursing career	Bad impression Horizontal Violence Poor performances Incompetencies Unequal opportunities Ideas of favouritism Lack of respect Incivility Harm	Language barriers Poor communication Second‐rate profession Racism Inequality Trust issues Culture and climate Verbal abuse Horizontal Violence	Organizational climate and culture Students' contributions Public impressions and views	The workplace aggression experiences	This theme focuses on the experiences of WPV that nurse educators have encountered from various perpetrators
Passion Adaptation Coping Adjustment Aspirations Dreams Motivations Professional development Success Still alive Fighting	Personal development Vision Stepping up Interdependence Collaboration Intrinsic motivation Change agent Independence Fighting Creative avenues	Extrinsic motivations Cooperation Mutual understanding Difference Change Keeping up Hospitality Survival Survivors Optimism	Passion for the nursing profession Aspirations to make a difference Motivations for personal and professional development	The keys to successful adaptation	This theme denotes how nurse educators triumphantly adjust, adapt, and cope with all the WPV experiences
Policies Advocacy Intervention Programs Activities for change Saudi Vision 2030 Connection Transformation Leading Transforming	Health strategies Nurse advocates Organizing Putting forward Brotherhood Mentorship Reflection Social media Health education Development Blueprint Sustainability	Nursing interventions Planning Coaching Moulding future nurses Heath teaching Alignment Mutual respect Recommendations Futuristic Action Plan	Promotions of nursing as a profession Creation of robust policy and advocacy programs Alignment with Saudi Vision 2030	The Recommendations for the future	This theme illustrates the nurse educators' suggestions for potentially decreasing and eliminating WPV

### 3.1 The Workplace Aggression Experiences

It pertain to the personal experiences of the nurse educators towards WPV designed to cause psychological or physical harm to a colleague or organization. Three categories emerged.

#### 3.1.1 Organizational Climate and Culture

It refer to the various aspects that may affect the WPV promotion within the organization, for example, ageism, social and cultural diversity, a lack of professional experiences, poor performance, incompetence, and unequal opportunities provided to each faculty member:

I am not an Arabic speaker. Most of the time, I feel left out on most occasions. However, it becomes normal at all. (P7 – Male, 36, Philippines, Single, Professor)

This is due to the fact that I lack numerous certifications and degrees. I perceive that I am afforded fewer opportunities in comparison to my peers. (P12 – Female, 35, Saudi Arabia, Married, Demonstrator)

#### 3.1.2 Students' Contributions

It reflect their actions, which contributes to WPV, including instances of disrespect, favouritism poor communication, and language barriers:

Some of the students are disrespectful. One time, I was reading my survey evaluations, and one student disliked me for being a foreigner and for not being able to speak their language. (P5 – Male, 44, Philippines, Married, Lecturer)

#### 3.1.3 Public Impressions and Views

It reveal how individuals outside the organization contribute to WPV, including a negative perception of the nursing profession, its perceived inferiority, racism, and sexism in the field:

A friend of mine told me that nursing is a female‐dominating profession and told me to leave the profession and find a new one that would fit my gender to give way to the female nurses. (P13 – Male, 46, Jordan, Married, Lecturer)

There is an impression that nurses are only assistants of the doctors and not capable of teaching students. (P14 – Female, 57, Egypt, Married, Professor)

Some people believe that men in nursing are rejects from the college of medicine. (P24 – Male, 37, Jordan, Single, Master)

### 3.2 The Keys to Successful Adaptation

It concern how nurse educators victoriously cope, adjust and adapt after experiencing WPV first‐hand. Three categories emerged.

#### 3.2.1 Passion for the Nursing Profession

It pertains to how nurse educators hold on and continue pursuing their careers in the academic settings:

I love teaching. It is my passion to teach future nursing professionals. No matter how challenging it is. I will do whatever it takes to fight WPV. (P4 – Female, 57, Sudan, Married, Professor)

I believe that being a nurse educator is an ultimate profession. We are the ones who mold the future nurses in the kingdom. We align everything we do for our students with Vision 2030. (P8 – Female, 37, Saudi Arabia, Married, Lecturer)

Despite my past experience of WPV, I want to help my colleagues and nursing students become the best they can be. It is our obligation to instill in the hearts and minds of everyone the essence of being a compassionate nurse, valuing respect and dignity without experiencing WPV. (P15 – Male, 52, Philippines, Married, Demonstrator)

#### 3.2.2 Aspirations to Make a Difference

It describe how nurse educators persevere in surviving by looking back to their lifelong goals and objectives to teach future nurses:

Having experienced WPV firsthand, I make sure to be keen on observing any forms of WPV. My colleagues should not have had the same bad experience I did. (P20 – Male, 38, Philippines, Single, Lecturer)

I always impart to my students not to engage in any forms of violence, whether inside or outside the university. Everyone should receive respect without any conditions. (P25 – Female, 59, Saudi Arabia, Married, Professor)

#### 3.2.3 Motivations for Personal and Professional Development

It illustrate how nurse educators continue to improve their nursing competencies despite their WPV experiences:

I always make sure that I have the required certifications to be competent to teach nursing education. Having these certificates gives me confidence and respect from people. (P14 – Female, 57, Egypt, Married, Professor)

As an expatriate, I am always up to date on my credentials. It protects me from any forms of WPV. (P19 – Female, 53, Philippines, Married, Lecturer)

I am determined to improve my personal and professional endeavors. I have witnessed how people respect a person with such a high educational background and certifications. (P21 – Female, 42, Saudi Arabia, Married, Professor)

### 3.3 The Recommendations for the Future

It pertain to the sustainable insights on how to eradicate and manage WPV in nursing education facilities based on their first‐hand experiences. Three categories emerged.

#### 3.3.1 Promotions for the Nursing Profession

It show how the nursing community can change the public's negative perception of the profession:

Occasionally, high school students invite me to speak at career days. I always make sure to promote nursing as one of the noble professions. (P8 – Female, 37, Saudi Arabia, Married, Lecturer)

One of our community initiatives in one of our major nursing courses is to invite high school students to our college for an ocular visit to our classrooms and facilities, giving them an idea of what nursing could offer them. If they are unable to attend, we conduct school visits to uphold community lecture services and promote nursing as a wonderful profession. (P12 – Female, 35, Saudi Arabia, Married, Demonstrator)

#### 3.3.2 Creation of Robust Policy and Advocacy Programs

It refers to the sustainable programs and activities that can be actualized to eradicate and manage WPV in educational settings:

I think educational facilities should implement an anti‐WPV program. This program will safeguard the welfare of the nurse educators from any forms of violence. (P1 – Female, 37, Saudi Arabia, Married, Lecturer)

The college has existing policies on WPV; however, we should strengthen, monitor, and evaluate these policies to ensure 100% implementation. (P17 – Female, 40, Saudi Arabia, Married, Professor)

#### 3.3.3 Alignment to Saudi Vision 2030

It denotes how micro initiatives from relevant nursing associations and organizations can be incorporated into the macro transformation program of the government:

One major objective of the health transformation strategy from the Saudi Vision 2030 is to envision the healthcare workers and educators' welfare to be excellent. Nowadays, WPV is rampant, and we should engage in continuous education to prevent its occurrence. (P22 – Male, 39, Saudi Arabia, Married, Lecturer)

## 4 Discussion

### 4.1 The Experiences of Nurse Educators Towards WPV

**FIGURE 1:**
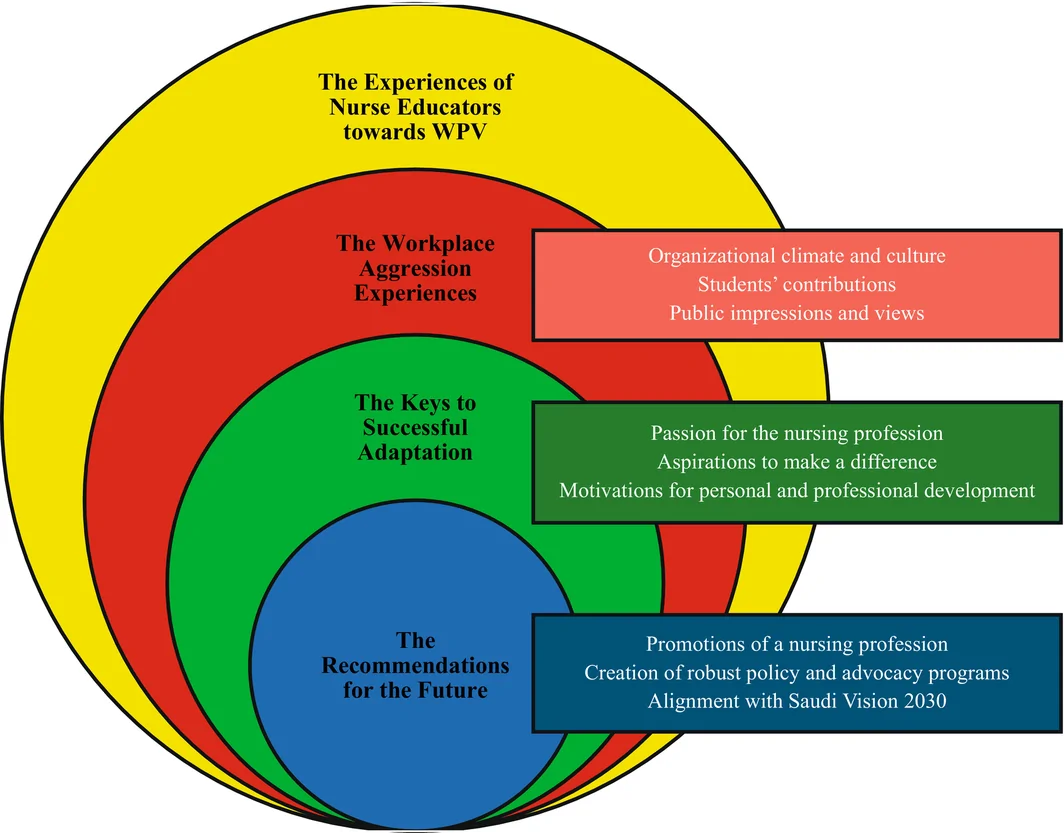
The experiences of nurse educators towards WPV.

WPV is a global issue with significant ramifications. Victims of violence may suffer physical, emotional, and mental harm, which can result in chronic stress. Academic professionals' morale and productivity might take a hit when they experience anxiety and fear, which can disrupt their ability to concentrate and focus on their duties and responsibilities. Absenteeism and turnover will result, as nurse educators and faculty members seek safer workplaces. Likewise, an organization's credibility, trustworthiness, and commercial relationships can take a turn for the worse following negative media coverage of a violent incident. In this descriptive study, the emergent themes and categories (Figure 1) reflect the nurse educators' experiences towards WPV, such as: (1) “the workplace aggression experiences,” (2) “the keys to successful adaptation,” and (3) “the recommendations for the future,” which will be discussed thoroughly in the next paragraphs.

“The workplace aggression experiences” stands as the first emerging theme. Some level of WPV has existed throughout history, in settings where people have worked together. The frequency of WPV is remarkably high. A high global prevalence has been documented and is expected to remain (Liu et al. 2019). Yet the impacts of horizontal violence are multifaceted. Professional nursing activities in several healthcare sectors are influenced by their consequences on individuals, groups, and organizations. Attributing it to the various personalities in the organization, this study focuses on the individual experiences of nurse educators in academic settings. This theme emerged from three categories: (1) organizational environment and culture; (2) student contribution; and (3) public impressions and views. In any case, healthcare professionals including nurse educators experience negative impacts from aggression, bullying, and incivility, which include a hostile work environment and physical and mental health issues like anxiety, insomnia, and depression (Vessey et al. 2010). Horizontal violence, aggression, bullying, and incivility are different; however, they are interrelated forms of undesirable behaviour and conduct that may evolve into WPV. The research team compiled straightforward definitions to establish a clear distinction between these four terms. Horizontal violence is passive, detrimental behaviour directed towards coworkers at the same level or within a group, such as insulting or mocking remarks in an indirect way, shouting, or exclusion from the group (Travaini et al. 2024). Aggression is defined as intentional verbal or physical acts oriented towards the objective of harming a person at work, including threats and physical assault (Schat and Frone 2011). Bullying is a repeated detrimental act taken by one person against another, with the intention of embarrassing, intimidating, or degrading them, frequently over a long period of time when there is a power imbalance (Tsuno 2022). Incivility denotes low‐intensity deviant behaviours that are disrespectful and injudicious, ambiguous in their intent to harm, yet subtle, undermining respect, and creating a hostile atmosphere (Zhang and Zhang 2026). The difference between the four is the level of intensity. Incivility and horizontal violence have lower intensity and are generally more subtle than aggression and bullying (most harmful). However, all four may facilitate an environment of workplace violence, as they increase stress, anxiety, and the possibility of major confrontation (Bambi et al. 2018).

Many registered nurses in the kingdom in all various fields of nursing are expatriates who do not speak and write Arabic language. As a result, they are having difficulty communicating with their colleagues, patients, and even students. The growing cultural and linguistic variety between local residents and nurses is not a problem exclusive to Saudi Arabia; it is a result of the high immigration rates in affluent nations like the US and Australia (Keatinge et al. 2002). A recent systematic analysis found that these communication impediments are common across many countries and are associated with lower overall healthcare quality. When managed properly, it can increase workplace productivity by encouraging more innovative thinking and a broader perspective. Another category that emerged is the students' contribution to WPV. How students act reveals problems, including prejudice, poor communication, language barriers, and a general lack of respect, all of which contribute to it. Classroom disrespect can take various forms, including but not limited to appearing uninterested or “playing around,” failing to take notes during a lecture, or taking over class debates. Conversely, “respect for one another and honoring differences” characterizes civility in nursing education (Ehsani et al. 2023). As per the participants, the most commont type of WPV among nurse educators and students is incivility (e.g., rude comments, disruption of the classroom or even threats), may be a major contributor to staff tension. Such actions increase stress, reduce job satisfaction, and increase interpersonal disputes. Faculty exhaustion, mental health and self‐efficacy are all directly impacted by student incivility, which may produce an atmosphere that is more likely to breed faculty‐to‐faculty confrontations (Gibson and Pohlmann 2024). Saudi Arabia and throughout the Arab region, teachers often report moderate levels of workplace bullying and incivility, and the lack of explicit anti‐bullying rules has been recognized as a critical predictor of these behaviours (Damra et al. 2026). Moreover, scoping reviews of incivility in higher education show that student incivility is one of the antecedents of broader incivility that can impair faculty job satisfaction and contribute to stress and psychological strain (Penconek et al. 2024).

Public views and attitudes continue to support the deeply established gendered prejudices that exist within nursing, specifically targeting male nurse educators. While many participants' narratives reveal the continuing prejudice against male nursing professionals, the analysis of these experiences is generally superficial. Men who are nurses or educators often face the public perception that nursing is intrinsically a “female” job, a notion that can be traced back to historical antecedents such as the Nightingale School of Nursing that established nursing as a domain for women (Karimi and Alavi 2015). Far from innocuous, such preconceptions function as potent social scripts regulating who is considered a valid member of the profession. Masculinity, which is generally defined as a cluster of attributes like assertiveness and authority, is often seen as being in contrast with the ideal feminine values of empathy and submission that nursing celebrates (Quinn et al. 2022; Ellemers 2018). Consequently, male nurse educators have been marginalized, authority challenged, and their presence considered suspicious or an oddity. These factors of marginalization are compounded by intersectional factors such as racism and the perception of inferiority, which may create a hostile work environment that may lead to WPV. When viewed through a hermeneutic lens, these gendered assumptions are not only artificial social constructions but also a means of justifying the exclusion and demoralization of men within the academic spaces of nursing. These ongoing stressors lead to professional distress and burnout and negatively impact the quality of instruction and emotional climate for students. When institutions fail or refuse to critically investigate and destroy such prejudices, they perpetuate a cycle of attrition wherein outstanding male educators are pushed from the profession, further entrenching the gendered gap and jeopardizing the future of the nursing profession.

Based on the nurse educators' first‐hand narratives, it is revealed that long‐term exposure to academic WPV in Saudi Arabia has significant and often under‐appreciated sequelae on their occupational wellbeing. Along with the well‐researched phenomena of burnout and turnover, recurrent episodes of hostility, incivility, or aggressiveness result in long‐term psychological suffering, such as persistent worry, emotional weariness, and post‐traumatic stress‐like symptoms (Park and Kang 2022). Participants typically claim that they continue to feel uncomfortable, hyper‐vigilant, and less worthy of themselves even long after the violent events. This emotional impact is generally shown in absenteeism, as teachers are compelled to take several sick days and/or purposefully avoid particular colleagues, offices, or campus sites associated with previous trauma. These coping strategies lead to a gradual withdrawal from professional and social obligations over time that threatens community sense and leads to emotional isolation (Matsuura 2026). In the most extreme cases, this exposure pattern results in faculty considering career attrition with statements about leaving academia altogether, or taking non‐teaching roles to avoid further psychological trauma and to regain personal wellness. These sequelae have detrimental effects on the overall mental health of the individual, stability in institutions, and talent retention in Saudi higher education and thus warrant further systematic investigation and purposeful intervention.

The second emergent theme is “The keys to successful adaptation.” Nurses' professional attributes contribute to the growth of nursing education and the promotion of nursing as a profession. Professionalism and competence are among the most important qualities that nursing professionals should possess to avoid engagement or having experiece any type of WPV (Pareek et al. 2023). Despite the obstacles they encounter on the job, nurse educators still have the important task of fostering nursing students apply what they have studied in the classroom to everyday clinical situations. In fact, the increasing complexity of ailments and diseases, the rising demand for healthcare professionals, and the globalization of healthcare services have all contributed to a critical shortage of nurses with advanced professional knowledge and skills. To train and improve nursing practitioners, educators with expert‐level nursing capabilities are needed (Satoh et al. 2020; Kuivila et al. 2020; Takase et al. 2020). Thus, it connects how essential nurse educators are in improving the nursing clinical practice. Clinical nursing instructors' exemplary pedagogical practices are the engine that propels their students' professional development (Zhang, Cai, et al. 2022; Zhang, Zhou, et al. 2022). To raise the bar for nursing education, faculty members teaching the subject need to have in‐depth knowledge of the most effective ways to help their students learn and retain information.

The nurse educators' clinical teaching practices are vital for improving nursing students' clinical competency, yet they are often not up to par (Gcawu and van Rooyen 2022). According to Saleem et al. (2020), nurse educators want to feel a connection to their work, which entails physically, mentally, and emotionally tapping into their strengths to do their jobs well. Additionally, nurse educators are more invested and expressively available during WPV when they operate in a physiologically safe and psychologically meaningful environment (Clark et al. 2022). Professionals in an encouraging workplace can maintain a healthy work‐life balance, receive regular professional development opportunities, have their efforts recognized, work together effectively, and communicate openly and honestly with one another. When cultivated correctly, it encourages people to show what they can do without worrying about repercussions and fosters an atmosphere of trust and safety (American Nurses Association 2021). To fulfil goals without endangering the learning environment for student nurses, nurse educators must continuously improve and streamline their services (Gawne et al. 2020). As a nursing professional, *it is a duty to stay competent in the profession you work in*, as mentioned in one of the publications of the American Nurses Association (ANA), “Nursing: Scope and Standards of Practice of 2021,” which establishes individual nurses' accountability. No matter the nursing setting, professional nurses have the challenging but necessary responsibility of keeping up with workplace standards, regulations, practice changes, and equipment updates. Throughout their careers, nursing professionals face several crises and other challenges that may compromise their ability to perform their jobs well. In addition, Farahmand et al. (2022) emphasized the need to assess nurses' competency to help them overcome challenges while safeguarding their emotional and physical well‐being. Similarly, he emphasized the importance of qualities such as leadership, teamwork, professionalism, education, informatics, interpersonal communication, and responsibility as the best characteristics for combating WPV. It is less likely to occur when nurse educators are personally and professionally competent and content.

“Recommendations for the Future” is the third and final emergent theme. The participants identified several problems in the current nursing profession, including the recruitment of high school graduates into the programs, inadequate nursing practices, individuality crises, and public ignorance about nursing. Elmorshedy et al. (2020) found that merely 32.5% of Saudis preferred receiving care from nurses. Most respondents (71.5%) expressed embarrassment about having a nurse in their family, suggesting a lack of value for the nursing profession. However, the study found that the median knowledge score was low, with an interquartile range of 50.0–66.7. Among the many socio‐cultural obstacles brought to light by the research are the following: the detrimental impact on social life (64.5%), the gender‐mixed milieu (34.9%), and the deferred matrimonial plans of female nurses (20.3%). This lack of knowledge, along with other perceived social and cultural obstacles, is what gives Saudi Arabs a bad impression of nurses. To address the poor perception of nurses among Saudis, it is important to understand these issues so targeted interventions can be developed and implemented.

Another crucial aspect of this theme is to instill in educators, not just nursing leaders and managers, the need to advocate for programs that eradicate WPV. It is indispensable for the nursing education sector to create robust policies and programs that ensure registered nurses across fields feel safe in exercising their duties and responsibilities (Qasem and Gillespie 2025). As stated in the first theme, failure to address it immediately leads to its integration into the organizational culture and climate. Policies and procedures should be an essential component of employee orientation, encompassing de‐escalation skills, identification and management of prospective perpetrators, and self‐defence measures (Wirth et al. 2021; Pagnucci et al. 2022; Pariona‐Cabrera et al. 2020). Furthermore, all nursing schools should implement WPV training programs for their students. Their ability to avoid and handle incidents depends on the training they receive, which provides them with the necessary information and practical skills (Clark et al. 2013). The sooner nurses understand the nature of WPV, the better prepared they will be to address its detrimental effects (Martinez and De Oliveira 2021).

Finally, to boost public health and the welfare of healthcare professionals, the Saudi government has emphasized the Vision 2030 Health Transformation Strategy. The United Nations (UN) has also reaffirmed that the 2030 Sustainable Development Goals will help bring about “healthy lives and promote well‐being for all ages” (Rahman and Qattan 2021). This initiative should grant nurse educators the right to healthy well‐being, free from all forms of violence, and always ensure their protection. To meet the healthcare transformation objectives of Saudi Vision 2030, it is important to go beyond general policy declarations and implement specific, institutional anti‐violence measures in healthcare settings. The first approach is to systematically tackle instances of WPV by introducing thorough systems of event reporting and rules of zero tolerance in the nursing education sector. These measures will not only discourage violent behaviours, but will also directly contribute to a safer and more contented staff which is vital to Vision 2030's objective to improve the quality of care. It is on this premise that nurse educators must turn to the keys to successful adaptation, including the roll‐out of resilience training programs, the setting up of peer support networks and the construction of open feedback loops between staff and leadership. The programs develop a flexible and motivated workforce that supports Vision 2030's emphasis on ongoing professional progress and a culture of innovation. Finally, future recommendations should be operationalized via ongoing, institutionalized anti‐violence measures such as deployment of quick response teams, persistent staff support, and periodic data‐driven assessments of workplace safety. Taken together, these targeted methods promote organizational responsibility and continuous improvement and convert the strategic objectives of Vision 2030 into a safer, more resilient, and sustainably changed healthcare system.

### 4.2 Limitations

One of the major limitations of the study is the researchers' biases in shaping data gathering and analysis. However, the research team practiced reflexivity throughout the process, following norms of scientific rigour such that the qualitative findings matched the criteria of authenticity, credibility, confirmability, dependability, and transferability. Another disadvantage was the lower sample size that limited the variety of experiences participants might have. Therefore, it was unable to generalize WPV experiences among nurse educators in the kingdom. Besides these limitations, the research was limited to one geographic location which may limit the transferability of results to other settings within the kingdom. Interviews conducted in English only may restrict the depth to which participants may communicate complex experiences, particularly for individuals for whom English is not their first language. Moreover, the study did not include the views of primary stakeholders such as students and university administrators, thereby restricting the scope of understanding of WPV dynamics inside the educational setting. Future research should address these limitations by employing multi‐region sampling, offering interviews in participants' native languages, and including a larger range of participants to provide a more comprehensive picture of WPV among nurse educators.

### 4.3 Implications for Nursing Education and Management

The occurrence of WPV in nursing education settings emphasizes the urgent need for nursing deans, program directors, and academic leaders to implement resilient, robust preventive policies and responsive actions. Practical and sustainable actions involve as follows: (1) setting up clear, confidential reporting systems that lead to reporting warning signs and enforce zero retaliation for reporting violence issues honestly, such as mobile applications, online portals and anonymous hotlines, ensuring all incidents are reported, recorded, and handled in a timely manner; (2) executing thorough and clear anti‐WPV written guidelines and protocols by managing constant policies' reviews, providing compulsory education with appropriate continuous professional development training activities to narrow safety gap at work areas and safeguarding coherent punishment and sanctions; (3) integrating simulation exercises and scenario‐based training on acquainting, analysing, and acting to more commonly encountered WPV during orientation programs for faculty members, staff, and students; (4) reinforcing leadership accountability and responsibility through crystalline communication, routine organizational climate surveys, and incorporation of evidence‐based WPV prevention outcomes in performance evaluations; (5) expanding the existing peer support networks and post‐incident mental health support and counselling after any WPV incident; in addition, to debriefing sessions to foster emotional support and practical guidance for the victims of violence; and (6) applying continuing monitoring systems in the educational facility—such as annual workplace climate assessments and feedback forums—will permit for initial detection of issues, difficulties, and complications, evaluation of intervention efficacy, and continuous improvement towards creating a healthy, safe, and supportive educational milieu.

In addition, future WPV research in nursing education needs to focus on strong empirical goals that will move the evidence base forward, not on broad advocacy. In particular, the researchers of this study propose the following approaches based on the results of this qualitative investigation: (1) Considering multi‐region sampling designs by conducting large‐scale cross‐sectional or longitudinal studies, including nursing professors and students from different regions of Saudi Arabia (northern or central). When possible, involve other GCC countries (Bahrain, Kuwait, Oman, Qatar, and United Arab Emirates) to allow comparison of prevalence, types, and risk factors for WPV in various macro and micro socio‐cultural and institutional settings; (2) Designing mixed‐methods program evaluation by integrating quantitative and qualitative surveys to assess real‐world adoption, acceptability, and impact of implemented anti‐WPV policies. Such evaluation approach would help identify both statistical effects and subtle contextual barriers or facilitators; (3) Conducting longitudinal assessments by following faculty and student cohorts to track long‐term outcomes of WPV prevention efforts such as retention, job satisfaction, and academic performance; (4) Instituting policy standardization initiatives by liaising with professional bodies to develop, pilot and empirically validate standards of anti‐WPV policies for all academic institutions. Furthermore, (5) comparative policy analysis might be undertaken to assess the Saudi frameworks against international best practices. The use of these empirical methodologies in future research would inform policy, improve staff and student safety, and improve nursing education culture in Saudi nursing education.

## 5 Conclusion

Various articles published in Saudi Arabia and in bordering Gulf Cooperation Council (GCC) countries, such as Bahrain, Kuwait, Oman, Qatar, and the United Arab Emirates, have reported the prevalence of WPV in clinical practice but none in the academic settings (Abdelmalik et al. 2025; Alhomoud 2025; Aljohani 2021). Nevertheless, the outcomes of this qualitative descriptive investigation, specifically the first emergent theme, horizontal WPV, show that the exploration of WPV in nursing education remains inadequate. The nurse educators' lack of self‐awareness about their occurrence led them to tolerate any form of abuse, whether physical, verbal, psychological, or financial. As a result, the violence becomes normalized and passed down through generations, thereby becoming a part of the organization's climate and culture. While it may be challenging to eradicate WPV in the nursing profession, especially in the academic setting, small steps can significantly improve the welfare of registered nurses and other healthcare specialists, as outlined in the health transformation strategy of Saudi Vision 2030. Therefore, there is no need for it to be threatening. Proactive measures can be used to improve, encouraging open communication in nursing schools. Educators, students, and everyone else involved in the academic workplace stand to benefit from a sense of security. Thus, “working as one” is one of the most significant factors in achieving a positive, productive workplace.

## Author Contributions

**Areej Al‐Otaibi:** software, data curation, writing – review and editing, methodology. **Reem Nasser Mohammed Al‐Dossary:** writing – review and editing, validation, methodology, conceptualization. **Ola Ibrahim Ramzi:** writing – review and editing, formal analysis, validation, data curation. **Jordan Tovera Salvador:** conceptualization, methodology, investigation, project administration, formal analysis, writing – review and editing, writing – original draft, validation, visualization, software, data curation, resources. **Abdulla Mohammed Ibrahim:** resources, data curation, project administration. **Friyal Mubarak Alqahtani:** software, data curation, writing – review and editing. **Sana Abdulkareem Almahmoud:** conceptualization, investigation, methodology, formal analysis, validation. **Neama Mohamed Kamel:** validation, methodology, formal analysis. **Basim Muzil Mohammad Al‐Anazi:** methodology, formal analysis, writing – review and editing. **Faisal Lafi Aldhafeeri:** validation, methodology, software. **Suzette Golez Cabonce:** methodology, investigation, visualization. **Ahrjaynes Balanag Rosario:** investigation, methodology, project administration, resources. **Maria Abigail Trinidad:** writing – review and editing, methodology, project administration, software. **Darwin Damsani Agman:** methodology, investigation, resources. **Eshtiaq Abdulaziz Alfaraj:** conceptualization, supervision, project administration, methodology, writing – original draft, software, data curation, formal analysis.

## Funding

The authors have nothing to report.

## Disclosure

During the preparation of the study's manuscript, the author(s) used Grammarly and QuillBot to correct English grammar, improve readability, and language clarity. The iThenticate application was used to check the similarity index and AI reporting. No AI tools were used for data collection, analysis, interpretation, or content production. All machine‐generated adjustments were meticulously evaluated to ensure accuracy and suitability. The author(s) reviewed the content and edited all machine‐generated adjustments as necessary and take(s) full responsibility for the accuracy and integrity of the work.

## Ethics Statement

Ethical approval was granted by the institutional review board of Imam Abdulrahman Bin Faisal University (IRB‐2023‐04‐136) on 22 March 2023.

## Conflicts of Interest

The authors declare no conflicts of interest.

## Data Availability

The authors have nothing to report.
